# The Public Health Impacts of Climate Change in the former Yugoslav Republic of Macedonia 

**DOI:** 10.3390/ijerph110605975

**Published:** 2014-06-05

**Authors:** Vladimir Kendrovski, Margarita Spasenovska, Bettina Menne

**Affiliations:** 1 WHO Regional Office for Europe, WHO European Centre for Environment and Health, Platz der Vereinten Nationen 1, Bonn 53113, Germany; E-Mail: menneb@ecehbonn.euro.who.int; 2 WHO Regional Office for Europe, WHO Country Office, The former Yugoslav Republic of Macedonia, Drezdenska 22, Skopje 1000, Macedonia; E-Mail: msp@euro.who.int

**Keywords:** adaptation, climate change, public health, vulnerability, impact assessment, strategy

## Abstract

Projected climatic changes for the former Yugoslav Republic of Macedonia for the period 2025–2100 will be most intense in the warmest period of the year with more frequent and more intense heat-waves, droughts and flood events compared with the period 1961–1990. The country has examined their vulnerabilities to climate change and many public health impacts have been projected. A variety of qualitative and quantitative methodologies were used in the assessment: literature reviews, interviews, focus groups, time series and regression analysis, damage and adaptation cost estimation, and scenario-based assessment. Policies and interventions to minimize the risks and development of long-term adaptation strategies have been explored. The generation of a robust evidence base and the development of stakeholder engagement have been used to support the development of an adaptation strategy and to promote adaptive capacity by improving the resilience of public health systems to climate change. Climate change adaptation has been established as a priority within existing national policy instruments. The lessons learnt from the process are applicable to countries considering how best to improve adaptive capacity and resilience of health systems to climate variability and its associated impacts.

## 1. Introduction

Climate change’s health risks are complex and wide-ranged: they include direct effects that mostly occur through increased frequency and intensity of extreme weather events, and indirect effects that are mainly induced by changes in major environmental, social and economic determinants of health [1]. Globally, there is increasing evidence showing the potential and emerging health impacts of climate change [2,3,4]. Risks are a product of complex interaction between physical hazards associated with climate change and climate variability on the one hand, and the vulnerability of a society or a social-ecological system and its exposure to climate-related hazards on the other. Vulnerability to climate change is defined as propensity or predisposition to be adversely affected [5].

Yet there is uncertainty about the timing, location, and severity of climate change. For Europe, the event with the most dramatic health impact attributed to climate change thus far, the heat-wave of 2003, occurred in the well-prepared industrialized world [6,7,8]. This illustrated the effects of an extreme weather event made on a relatively unprepared public health sector [9], and by high levels of both population exposure and susceptibility [10]. 

This paper reports the findings of the national health vulnerability, impact and adaptation assessment of climate change (*(h)*VIA) and describes the process and results of the development of the national health adaptation strategy and action plan (N(H)AP). The former Yugoslav Republic of Macedonia was one of seven countries involved in a pilot project “Protecting health from climate change in southeast Europe, central Asia and the Russian north”, which aimed to protect health from climate change through addressing adaptation, strengthening health systems and building institutional capacity. A technical and comprehensive assessment of vulnerabilities, impacts and adaptation options was undertaken in each of the seven countries. The aim was to identify not only the type, scale, nature and direction of climate variability exposure and health risks, but also the adaptation measures currently in place. The data and information collected were used to establish the evidence base to define the scope of national adaptation [11].

## 2. Experimental Section

A national framework was established to oversee activities: this included a national multisectoral steering committee and a technical working group, which defined the scope of the national adaptation plan, methods, peer review process, and process of defining priorities. A project team carried out a baseline scoping exercise to identify readily available information and data on the climate associated health exposures and risks to population health. An overview of the specific objectives and methods is given in Table 1. The scoping exercise and the vulnerability and impact assessment were conducted by a team from the Institute for Public Health, under the direction of the national steering committee. 

**Table 1:** Specific objectives and methods.

Objectives	Methods Used
To assess the health impacts and vulnerability to climate change	Qualitative and quantitative epidemiological methods: Literature reviews Stakeholders interviews Focus groups Time series and regression analysisScenario based assessment Damage and adaptation cost estimation for heat-waves
To develop a national health adaptation strategy	Public health approaches supported by: Stakeholder engagement plan International and national dialogue workshops Health systems assessment

### 2.1. Health Vulnerability and Impact Assessment ((h)VIA)

The *(h)*VIA used the methods developed by World Health Organization and others [12,13]. It utilized a range of qualitative and quantitative methodologies, reflecting data and information availability and the nature of the challenges under review, as shown in Table 2. Additional data were collected through case studies, stakeholder interviews and focus group discussions with representatives of all sectors responsible for climate change adaptation and mitigation. Where specific vulnerabilities had been identified, in-depth analyses were performed to improve understanding of the scope and scale of the exposures and risks. This included investigation of the influence of heat-waves on mortality, the risks associated with salmonellosis, the presence of the vector *Aedes albopictus* and the seasonal onset of pollen and aeroallergens in the city of Skopje [14]. 

Analysis of the data was carried out through time series and regression analysis for selected health outcomes, scenario based impact assessment and a damage and adaptation cost estimation for heat-waves (Table 2). At national and local level, climate projections have been used to inform a basic review of the types of potential impacts, with limited evaluation of the capacity, capability and resilience to respond to these impacts. 

**Table 2:** Vulnerability and impact assessment approach for climate sensitive diseases and conditions.

	Extreme Events: Heat/Cold	Extreme events: Flood/Drought	Infectious Diseases	Air Pollution/ Allergies and Pollen
**Assessment method **	Time series analysis and Poisson regression analysis on heat-related mortality in Skopje [15,16]. Excess winter mortality, nationwide (1994–2008) [16]. Scenario based assessment (A1B and A2) [14]. Damage and adaptation costs analysis of heat-waves in Skopje [17].	Systematic literature reviews by key words: flood, drought, health, climate change, R. Macedonia [16]. Semi-structured Interviews with national key stakeholders. Focus group and action research with stakeholders.	*Salmonella* human cases time series and Poisson regression analysis (1998–2008) in 5 cities of the former Yugoslav Republic of Macedonia [18]. Scenario based assessment (A1B and A2) [18]. Systematic literature review by key words: mosquito, R. Macedonia, climate change [19]. Field investigation in 6 sites for presence of dengue-mediating mosquitoes [19].	Regression analyses [20]. Quantifying the onset of flowering, maximum and end of the length of seasons for 9 types of pollen during the vegetation period of 1996–2009 [20]. Quantifying the current and projected future burdens of temperature [20,21].
**Data and information sources**	Meteorological data (National Hydro-meteorological Institute) Daily counts of deaths (State Statistical Office) Damage and adaptation toolkit software (WHO pilot project) Scenarios developed for the country (22)	Crisis Management Centre Protection and Rescue Directorate Ministry of Environment and Physical Planning	Meteorological data (National Hydro-meteorological Institute) Weekly counts of human *Salmonella* confirmed cases (National Institute for Public Health) Scenarios developed for the country (22)	Air pollution data (Ministry of Environment and Physical Planning) Meteorological data (National Hydro-meteorological Institute) Daily distribution of 9 pollens (National Institute for Occupational Medicine)

### 2.2. Health Adaptation Strategy Development Process

As no specific guidelines were available at the start of the project, the WHO Regional Office for Europe developed a public health framework and a step-wise approach [11]. For each step, guiding questions were debated within the national steering committee and amongst stakeholders. 

**Figure 1:**
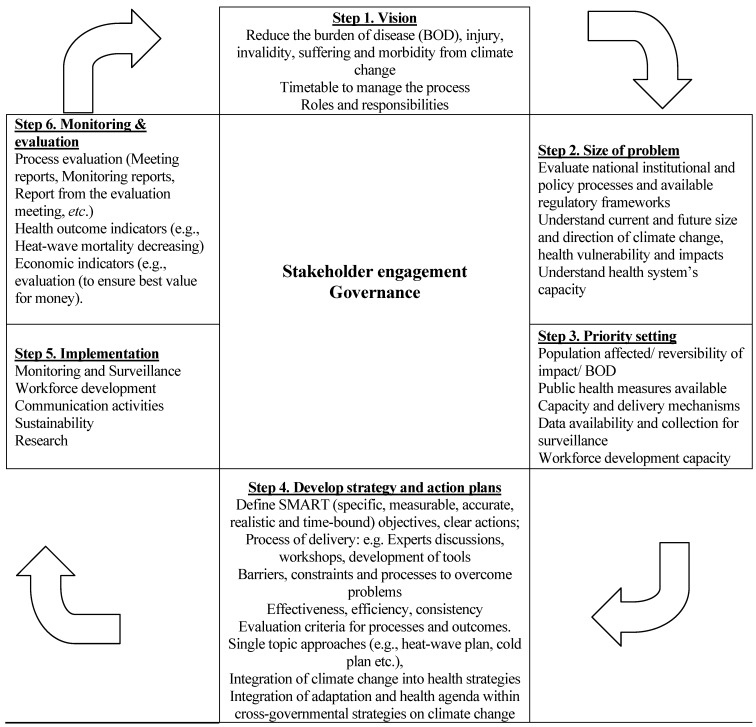
.Framework for the development of a climate change health adaptation strategy.

Note: Source: Adapted from WHO public health adaptation framework [11].

A three-day workshop was held in 2009 to identify general priorities and provide strategic direction for the development of a climate change health adaptation strategy, using the WHO public health approach as shown in Figure 1. The workshop was attended by representatives from the Ministry of Health, Institute for Public Health, Ministry of Environment and Physical Planning, Hydrometeorological Institute, Crisis Management Centre, Protection and Rescue Directorate, emergency medical services, public health centres, Institute for Occupational Medicine, World Health Organization, Red Cross and other nongovernmental organizations (NGOs).)

Key factors considered at the workshop included: (a) the size and the nature of the problem related to each of the priorities, (b) effective interventions, (c) the goal and objectives of the strategy for each of the priorities, (d) involvement of institutions and responsibilities, (e) indicators, and (f) financial implications of each proposed interventions. For the development of the health adaptation plan, three multisectoral working groups were established, with specific responsibilities for assigned priority areas (air quality, extreme weather and infectious diseases). The criteria used for detailed development of the strategy included the approximate costs of proposed interventions, benefits of interventions to health and other sectors, feasibility to implement the interventions within existing services or systems, potential harm from any intervention, and potential obstacles and opportunities for implementation. Comments received at the workshop were incorporated into the draft strategy, which was then sent to selected local experts for peer review and revision. 

## 3. Results and Discussion

### 3.1. Results from the (h)VIA

Compared with the period 1961–1990, the projected changes for the country for the period 2025–2100 will be most intense in the warmest period of the year with more frequent and more intense heat-waves and droughts. Scenarios A1B and A2 are used for temperature and precipitation projected for the period to 2100 [14,22].

Around 10% excess winter mortality has been observed on average during the period 1996–2000 in the country, which has about 2,050,000 inhabitants. Mortality within the city of Skopje displayed a marked seasonality, with peaks in the winter relative troughs in the summer [15]. The most striking anomaly was in 2007, when the government declared a nationwide heat-wave emergency. During the summer of 2007, daily temperatures reached 43 °C and caused more than 1000 excess deaths at national level (compared to the averages of 1994–2008). In Skopje, the capital, with around 600,000 inhabitants, the July temperatures in 2007 were 3.4 °C above the monthly average and deaths were 16.5% higher than the average between 1994–2008 [16].

The *(h)*VIA process identified a number of specific climate sensitive conditions and diseases where adaptation is required. The impacts of severe climate-related hazards are already manifest in the country and some will increase in scale. Major heat-waves, floods and droughts have led to deaths and human suffering, social disruption and a substantial burden on health systems. The direct health effects of heat waves could become a significant problem, especially with more than 60% of the population living in the cities and urbanization increasing. The heat cut-off point for the maximum temperature for Skopje, the capital, was 30.8 °C. Under heat-wave conditions, with an increase of 1 °C above heat cut-off point for Skopje, the total mortality increased by 4.8%. Increased mortality during heat waves is higher among elderly people with chronic diseases, and cardiovascular and respiratory diseases [14,16]. A national study showed that it is possible to compare the costs of adaptation with the damage costs of the increase in disease cases and deaths caused by severe unavertable climate-related hazards. Based on the economic estimation the annualized costs of heat-health adaptation measures were estimated at 12 million denars (MKD) compared to health damage costs of MKD 170 million per year [17]. 

Existence of the *Aedes albopictus* mosquito as a potential vector for some diseases was not found during the investigation carried out in 2010 [19]. Changes in pollen seasonality have also been observed in the country for the 1996–2009 period [20]. 

Skopje is situated in a valley and the river Vardar crosses the city, causing higher humidity during the winter. An atmospheric anticyclone contributes to atmospheric temperature inversion and consequently, worsening ambient air pollution with PM_10,_ and increasing daily admissions of patients with cardiovascular diseases [23]. 

Food safety is compromised by higher temperatures, with several studies confirming and quantifying the effects of high temperatures on common forms of food poisoning, demonstrating an approximately linear increase in reported cases of *Salmonella* with each degree increase in weekly temperature [18,24,25]. 

In addition, changes in the distribution of vectors are likely to result in re-emergence and increased incidence of vector-borne diseases. Recently, some vector-borne diseases sensitive to climate variations (e.g., malaria) have been reported in neighbouring countries, while other climate-sensitive vector-borne diseases have occurred in the former Yugoslav Republic of Macedonia (e.g., West Nile fever) [26,27]. The scale of this challenge calls for effective partnership work and intersectoral engagement. This is a shared agenda, not a health services agenda. Continuous epidemiological surveillance and advanced early warning systems need to be supported by robust intersectoral communication, a shared vision and support for effective action. 

The *(h)*VIA showed that a number of current measures, policies and strategies needed to be revised or strengthened to respond to current and projected levels of risks from climate change, e.g., strengthening the surveillances and monitoring of infectious diseases or capacity building among health professionals. The *(h)*VIA process identified a number of specific climate sensitive conditions and diseases where adaptation is required. The results are summarized in Table 3. The impacts of climate variability on health are already evident. Over 1,000 excess deaths in the heat wave during the summer of 2007 [12] were observed in the country, together with changes in pollen seasonality [20,21] and changes in the spatial distribution of some infectious disease vectors [28]. 

**Table 3:** Results from *(h)*VIA.

Climate Sensitive Diseases and Conditions	Impact: Current	Impact: Projected
Floods/Droughts	Catastrophic floods in past resulted in serious damage to residential areas and infrastructure as well as to the water supply and sewage systems. The weather conditions in 2007 led to one of the most extreme droughts since meteorological records began in the country [16]	Projections indicate that the combined impact of flooding events over the next century will likely affect about 4,050 households in the Skopje valley and about 1,750 households in the Pelagonia valley within the next 100 years Droughts are very likely to increase in number and severity, resulting in loss of agricultural land, with both nutritional and economic consequences [16]
Heat/Cold Extreme events	The climatic variables such as mean winter ambient temperature are found to be positively associated with levels of relative excess winter mortality in the country. Hot weather has a stronger impact on human mortality than cold weather. A heat-wave was defined as a period when maximum apparent temperature and minimum temperature are over the ninetieth percentile of the monthly distribution for at least two days. Under heat-wave conditions, an increase of temperature of 1 °C above the heat cut off point for Skopje (30.8 °C) leads to an increase in mortality of 4.8% [13]. Economic analysis of the costs of adaptive action was also performed. This found that the total heat-wave costs were approximately twice those of climate change-attributed costs, at approximately MKD 170million per year, or € 2.6 million [17].	In a warmer future climate, very likely there will be more intense, more frequent and longer-lasting heat-waves in the country [29]*.*
Infectious diseases	Seasonal patterns of *Salmonella* infections, with a peak in the summer months after the peak of temperatures were detected. For Skopje, an increase in the weekly temperature of 1 °C above detected threshold of 17.9 °C was associated with a 2.8% increase in salmonellosis cases [18]	The projections showed that the whole Balkan Peninsula in the future is very likely to be suitable for establishment of *Aedes albopictus* [30].
Air quality	Air pollution may compound the effects of high and low temperature morbidity on daily ambient levels of PM_10_ (μg/m^3^) and ozone (μg/m^3^) [23]	Increase of 10 μg/m^3^ of PM_10_ above maximum permitted values (50 μg/ m^3^) will likely result in increasing the daily admission of patents with cardiovascular diseases in Skopje by 12% [23].
Allergies and pollen distribution	The prevalence of standard pollen allergens in Skopje shows an increase from 16.9% in 1996 to 19.8% in 2009 [20]. Allergic risk increases in 3 main periods: early spring, spring and summer, in which standard pollen allergens are the main cause of allergies during those months [20].	The impacts of climate change, via increasing temperature in the next decades on aeroallergens, and in particular pollen, will likely include impacts on pollen production and pollen season (onset of flowering, maximum and end of the seasons) [20].

Broad stakeholder engagement and a participatory approach towards adaptive management result in and support more interdisciplinary implementation efforts [31]. 

The evidence collected through the *(h)*VIA process visibly demonstrated that climate change is likely to have both direct and indirect impacts on the health of the country’s population. It highlighted that these impacts are happening now, and that they do not occur in isolation, but exist alongside and potentially exacerbate existing challenges to health, social, environmental and economic systems. Climate-related changes in air quality and pollen distribution are expected to affect several respiratory diseases. In coming years more intense, more frequent and longer-lasting heat-waves in Skopje are expected to have a stronger impact on human mortality. 

About 10% of the population still lacks access to clean and safe water, be it for drinking or for meeting their basic needs. Droughts, poor irrigation, and the damage that flooding will cause to existing sewage systems will exacerbate this [16]. Systematic assessments of the resilience of water supply and sanitation systems to climate change and inclusion of its impacts in water safety plans are needed.

### 3.2. Climate Change Health Adaptation Strategy

On the basis of the *(h)*VIA, the climate change health adaptation strategy [32] was developed and endorsed by the national government in February 2011 with the following priorities:

- raising awareness of climate change and the effect on health;
- identifying, registering and monitoring risks connected with climate change and their influence on people’s health, and
- improving the health sector’s promotion and prevention activities.

The general goal of the strategy is to plan climate change adaptation measures for the health sector in order to prevent and/or overcome both existing and future risks and to respond promptly to specific risks and problems for people’s health that are expected as a result of climate change in the country (Table 4).

**Table 4:** Proposed adaptation measures for the national health adaptation strategy.

Climate Sensitive Diseases and Conditions	Adaptation Measures
Heat/Cold Extreme events	National Heat Health Action Plan in place since 2010 as a pilot (evaluated December 2012) [28]. National Cold Health Action Plan endorsed in 2012 [33].
Flood/DroughtExtreme events	Establish an integrated, efficient and effective approach for prevention, early warning, management to overcome the effects of climate change connected to floods and fires [32]; Other plans; Municipality action plans; Flood risk plans; Effective operational strategies to improve irrigation required.
Infectious diseases	Strengthening existing epidemiological surveillance, reporting, monitoring and analysis of communicable diseases that are transmitted by water, food and vectors. Education of the general public on the benefits and necessity of: -implementation of adaptation measures such as Hazard Analysis Critical Control Point (HACCP) system; -disinfection and extermination of rats and insects. Undertake preventive disinfection and pest control (extermination of rats and insects) in educational, social and health institutions [32]
Air quality/Allergies and pollen	Overcome the climate change health consequences connected with air pollution and cold weather during winter by establishing effective control and specific preventive measures. Continuous pollen monitoring and reporting on the type and concentration of pollen grains in the atmosphere on a daily basis [32].

Other areas addressed by the national health adaptation strategy include: adapting the health care infrastructure (hospitals, nursing homes) to be more resilient to the effects of heat, fires and floods; development of local “Safety Hospital” plans for coping with disasters; and increasing awareness of how people can adapt to changes in climate. The development of the strategy publicised the need for greater emphasis to be placed on climate change and its impacts; the need for governments to focus on this problem; and measures individuals can take to mitigate the effects of climate change on their health [32]. 

The *(h)*VIA provided a firm baseline for building cross-sectoral adaptive capacity. It identifies key risks and exposures, and recommends steps which can be taken to engage communities in the development of resilience and capacity to prepare for changing climate conditions and extreme weather events. By identifying areas where vulnerability is particularly high—threats that exhibit distinct climate sensitivity—it provides a good example of the ways in which *(h)*VIA processes can help clarify where efforts to increase adaptive capacity should be focused, and ensure that efforts to address climate change are appropriately incorporated into broader health, social, environmental and economic policies and strategies. For example, poor communities, without access to good health protection and support from the social sector, are more susceptible to the negative health effects that arise from changes in climate and the living environment. Efforts to improve resilience to climate change can therefore affect both the health and social determinants agendas, and have broader economic impact. However, ensuring that this evidence and commitment to action is translated into practical on the ground action remains a fundamental concern. 

This national assessment has shown that public health institutions at all operational levels will need to consciously modify their approaches to both science and practice in anticipation of climate change health impacts. The international literature makes a strong case for outlining climate change as a public health concern, and advancing the public health community’s awareness [34,35]. 

## 4. Conclusions

Institutional learning at multiple levels has been key to increasing adaptive capacity, and adaptive management. The development of a national strategy for health sector adaptation due to climate change [32] was identified as a high priority and, along with an increased focus on learning, modelling, and adaptive management, will help increase the resilience of local public health systems. The country is making significant strides towards developing the adaptive capacity to improve climate resilience by building an integrated, efficient and effective public health approach. 

Identifying appropriate methods for conducting the national impact assessment was challenging, with limited available data and human capacity resources. For many health impacts, climate change is not a trigger problem but one that will develop over decades or longer, or impact indirectly. 

Whilst building adaptive capacity remains an ongoing concern, many of the successes in this assessment have already begun to have an impact. Throughout the process, a number of tools were utilized which allowed practitioners to organize information on the hazard and population at risk in order to prioritize responses. These successes provide a robust and ongoing foundation for future collaboration and activity. Whilst there have been specific project benefits, such as increasing the awareness among health professionals of the health impacts of climate change, and the development of the Heat Health Action Plan and Cold Health Action Plan, the broader benefit has been the increased engagement of key stakeholders and policy-makers with this agenda. Before the process was initiated, climate change and health topic was largely marginalized, with activity happening in silos. 
